# Inactivation of Influenza A Viruses (H1N1, H5N1) During Grana-Type Raw Milk Cheesemaking: Implications for Foodborne Transmission Risk

**DOI:** 10.3390/v17121535

**Published:** 2025-11-24

**Authors:** Ana Moreno, Stefano Pongolini, Giuseppe Merialdi, Giovanni Cattoli, Calogero Terregino, Nicola Santini, Stefano Benedetti, Luisa Loli Piccolomini, Anna Padovani, Alfonso Rosamilia, Giovanni Loris Alborali, Paolo Daminelli

**Affiliations:** 1 Istituto Zooprofilattico Sperimentale of Lombardy and Emilia-Romagna, Via Bianchi 9, 25124 Brescia, Italy; stefano.pongolini@izsler.it (S.P.); giuseppe.merialdi@izsler.it (G.M.); alfonso.rosamilia@izsler.it (A.R.); giovanni.alborali@izsler.it (G.L.A.); paolo.daminelli@izsler.it (P.D.); 2 Istituto Zooprofilattico Sperimentale delle Venezie, Viale dell’Università 10, 35020 Legnaro, Italy; gcattoli@izsvenezie.it (G.C.); cterregino@izsvenezie.it (C.T.); 3 Ministry of Health, Via Giorgio Ribotta, 5, 00144 Rome, Italy; n.santini@sanita.it; 4 Regione Emilia-Romagna, Settore Prevenzione Collettiva and Sanità Pubblica, Viale Aldo Moro 21, 40127 Bologna, Italy; stefano.benedetti@regione.emilia-romagna.it (S.B.); luisa.lolipiccolomini@regione.emilia-romagna.it (L.L.P.); anna.padovani@regione.emilia-romagna.it (A.P.)

**Keywords:** highly pathogenic and low pathogenic avian influenza virus, H5N1 HPAIV clade 2.3.4.4b, Grana-type raw milk cheese, virus inactivation, pasteurization, food safety, cheesemaking, viral persistence

## Abstract

The detection of H5N1 highly pathogenic avian influenza virus (HPAIV) in lactating dairy cattle in the United States, with high viral titers in raw milk, has raised concerns about zoonotic transmission through unpasteurized milk and dairy products. While viral inactivation during pasteurization is documented, data on persistence in raw-milk cheeses remain limited. This study evaluated the survival of avian influenza viruses (AIVs), both low pathogenic (LPAIV, H1N1) and highly pathogenic (HPAIV, H5N1), during the production and ripening of Grana-type hard cheeses from raw cow’s milk. Experimental cheesemaking was carried out with milk artificially contaminated with A/duck/Italy/281904-2/06 (H1N1; 10^7.75^ EID_50_/mL) or A/duck/Italy/326224-2/22 (H5N1 clade 2.3.4.4b; 10^6.75^ EID_50_/mL). Cheeses were manufactured under Parmigiano-Reggiano standards and ripened 30 days at 5–6 °C. Viral detection in finished cheeses was performed using inoculation in specific-pathogen-free embryonated chicken eggs (SPF-ECEs), hemagglutination (HA) assay, and monoclonal antibody-based ELISA. No infectious virus was detected in cheese samples after two blind passages in SPF-ECEs. Both HA and ELISA tests were negative, indicating complete viral inactivation. These results demonstrate that Grana-type cheese processing, including cooking, acidification, and ripening, effectively inactivates LPAIV and HPAIV. Findings support the microbiological safety of raw-milk hard cheeses regarding AIV, contributing to risk assessment and food safety policies.

## 1. Introduction

Highly pathogenic avian influenza (HPAI) continues to pose a substantial threat to animal and public health globally, particularly affecting poultry and wild birds [1]. Among the various HPAI subtypes, H5N1 influenza A virus (HPAIV) remains of particular concern due to its zoonotic potential and continued global spread. The currently circulating H5N1 viruses derive from the A/goose/Guangdong/1/1996 (GsGd) strain, first identified in China in the late 1990s. These viruses have undergone extensive reassortment, giving rise to multiple genetic variants. Since October 2020, H5N1 viruses of clade 2.3.4.4b have been detected across Europe, following reassortment between H5N8 and wild bird-origin N1 viruses. From autumn 2021 onward, clade 2.3.4.4b viruses have become dominant and have demonstrated efficient geographic spread via migratory birds. Notably, these viruses exhibit a high capacity for reassortment with co-circulating avian influenza A viruses and the ability to infect mammals, especially bird-feeding species [1]. In late 2021, clade 2.3.4.4b was reported in Canada, marking its first detection in the Americas [2]. The strains belonging to this clade, genetically similar to European viruses, underwent reassortment with low-pathogenicity influenza viruses endemic to North American bird populations, resulting in new genotypes with distinct internal gene constellations.

In March 2024, the first case of H5N1 infection in cattle was reported in Texas, United States (US), challenging previous assumptions about the low susceptibility of cattle to influenza A viruses [3,4]. Subsequent investigations revealed widespread infection: by 10 October 2025, 1081 confirmed outbreaks had been reported in dairy herds across 18 states in the US [5]. Whole genome sequencing confirmed these viruses as belonging to clade 2.3.4.4b, genotype B3.13, containing four gene segments of North American origin (NS, PB1, PB2, NP) and four of Eurasian origin (HA, NA, MA, PA) [3,6]. Furthermore, a second genotype D1.1, which predominated in North American wild birds during the previous winter spilled over into the U.S. dairy cattle population in early 2025 [7].

Clinical signs reported in H5N1-infected cattle have included respiratory and gastrointestinal symptoms, decreased milk production, and alterations in milk appearance, often colostrum-like, accompanied by dehydration and lethargy [6,8]. Among these, changes in milk quality and production volume are the most readily observed, as they are detected during routine milking procedures. Tests on raw milk samples from symptomatic animals revealed, quite unexpectedly, extremely high viral titers—reaching up to 10^8^ TCID_50_/mL—and a strong affinity of the virus for mammary gland tissue [9]. These unusually high viral loads, coupled with documented cases of lethal H5N1 infections in farm cats that ingested raw milk from infected cows, have raised urgent concerns about the safety of the US dairy supply.

Following the surge in outbreaks among dairy cattle and poultry, several human cases have been reported in the US, primarily among agricultural workers, with 70 confirmed infections and one death [10]. Although H5N1 was widely detected in birds and mammals across nearly all regions globally in 2024 [11], the total number of human cases remained relatively low (*n* = 85) [12]. Importantly, approximately 80% of these cases occurred in the US—the only country to report widespread infections in lactating cattle—underscoring the need to assess the potential for bovine-to-human transmission [13].

Although US regulations mandate the disposal of abnormal milk prior to pasteurization [14,15], asymptomatic or preclinical infections could result in contaminated milk entering bulk storage. A study conducted in 2024 detected influenza A virus RNA in 20.2% of pasteurized milk and dairy samples via quantitative real-time RT-PCR (qRT-PCR), though no infectious virus was recovered, supporting the efficacy of pasteurization in inactivating the virus [16]. Nonetheless, high viral loads in raw milk and potential exposure through unpasteurized dairy products continue to pose a public health concern.

Hard cheeses produced from raw milk, such as Grana-type cheeses, are of particular interest. These cheeses, exemplified by Parmigiano-Reggiano and Grana Padano, are widely consumed and exported. Their traditional manufacturing process involves raw milk, natural skimming, the addition of natural whey, curd cooking, and extended aging. To date, limited data exist on the behavior and inactivation of influenza A viruses during traditional cheesemaking, especially in European-style products.

Although it is known that highly pathogenic avian influenza viruses (HPAIVs) are sensitive to heat, acidity, and salinity [16,17], data on their survival and inactivation during the production of hard cheeses from raw cow’s milk are limited. Controlled experimental studies are therefore needed to assess the survival and inactivation of HPAIV during the entire production and ripening process of hard cheeses made from raw cow’s milk, in order to provide useful information for both food safety regulations and zoonotic risk mitigation. The aim of this study was therefore to investigate the persistence and inactivation of two influenza A subtypes (low pathogenic H1N1 and highly pathogenic H5N1) during the production of Grana-type cheeses. The cheesemaking process was simulated in a controlled laboratory setting, reproducing key steps including raw milk usage, natural whey inoculation, curd cooking, and storage under whey. Preliminary trials were conducted to validate virus detection and isolation methods in cheese matrices and to assess virus stability in ultra-high temperature (UHT) milk. These trials provided essential information to guide the design of the main experimental study.

## 2. Materials and Methods

### 2.1. Biosafety and Biosecurity

All procedures related to the handling and propagation of the H1N1 LPAIV, cheese production and processing, and virus isolation in ECE were performed in the BSL-2 laboratories of the Department of Virology at IZSLER, under strict biosecurity conditions and class II type B2 laminar flow hoods equipped with HEPA filters, to ensure operator safety. Procedures involving the handling of the H5N1 HPAIV, including viral isolation and cheese production as described above, were conducted in the BSL-3 plus laboratories of the same department, also in compliance with strict biosecurity measures and under class II type B2 laminar flow hoods with HEPA filters, to ensure operator safety.

### 2.2. Viruses

Two influenza A viruses were selected for the study: one highly pathogenic and one low pathogenic. First, we used a low-pathogenic H1N1 virus (A/duck/Italy/281904-2/06) to establish procedures for viral titration and virus isolation in milk and cheese, using a controlled-access BSL-2 laboratory. Among the low-pathogenicity viruses, we selected a strain isolated from wild birds in our region, belonging to one of the most frequently detected subtypes (H1N1), in order to ensure greater relevance to the study than if we had used a virus not circulating in our country. For the high-pathogenicity virus, we selected the H5N1 strain (A/Mallard/Italy/326224-2/22VIR9647-1/2022, EPI_ISL_19462780, EA-2021-AB), belonging to clade 2.3.4.4b, which is the same H5 clade responsible for infections in cattle in the United States [3].

### 2.3. Validation of Sample Preparation

The raw cow’s milk came from a dairy farm whose bulk milk had been tested for influenza A genome using real-time RT-PCR targeting the M gene [18], with negative results. In addition, each batch of milk resulted negative for influenza A genome before starting the trials. The physical, chemical, and microbiological properties and parameters of the milk samples were analyzed in the laboratories of the Italian Reference Center for milk quality (IZSLER).

Viral stock titrations were performed using 10-fold dilutions in PBS and inoculation into SPF chicken embryos to calculate the EID_50_/mL. Dilutions of the viral stocks were prepared both in PBS and directly in UHT and raw bovine milk to obtain a viral titer of 10^7^ ± 0.25 EID_50_/mL, taking into account possible minor variations in titer due to differences among viruses and matrices. H1N1 LPAIV viral titrations were performed in contaminated matrices such as PBS and UHT milk for preliminary tests and PBS and raw milk for trial 1 to determine whether different matrices affected viral titers.

To assess the suitability of cheese homogenates for virus isolation and titration in specific-pathogen-free embryonated chicken eggs (SPF-ECEs), a preliminary test was conducted using two 1 g portions of Grana cheese following the procedure described by FDA [19] with some minor alterations to accommodate the specific experiment’s needs. One sample served as a negative control, while the second was spiked with 100 μL of an H1N1 viral suspension (hemagglutination [HA] titer: 1:128 per 25 µL). The positive control was prepared following ISO 15216 protocols [20] as a process control by adding the virus to the sample to measure recovery efficiency and monitor potential inhibition. Both samples were cut into small pieces using sterile scissors and transferred into 15 mL Falcon tubes containing three 4.5 mm stainless steel beads and 10 mL phosphate-buffered saline (PBS) supplemented with antibiotics (final concentration: 10% *w*/*v* homogenate). In trials 1 and 2, conducted to verify the inactivation of influenza A virus during the production of Grana-type cheese, the following sampling protocol was used. For each batch of contaminated milk, three 1 g samples were collected from three different internal points of each cheese at the end of the experiment. For each batch of uncontaminated milk, a single 1 g internal sample was collected from each cheese at the end of the experiment.

Samples were homogenized using a TissueLyser II (Qiagen Italia, Milan, Italy) at 30 Hz for 5 min and then centrifuged at 3000× *g* for 10 min at 4 °C. From each sample, two 1 mL aliquots were prepared: one filtered through a 0.45 µm syringe filter, the other left unfiltered. All aliquots were inoculated into SPF-ECEs as described in Section 2.2. The matrix’s potential toxicity and its compatibility with virus detection in eggs were evaluated after 4 days of incubation at 37 °C.

### 2.4. Virus Isolation and Titration in Specific-Pathogen-Free Embryonated Chicken Eggs (SPF-ECEs)

Before inoculation, SPF-ECEs were candled to confirm embryo viability, and the air sac line was marked. The eggshell surface was disinfected with iodine tincture before and after puncturing. For virus isolation, 200 µL of each sample was inoculated into the allantoic cavity of 9–10-day-old SPF-ECEs, using at least four eggs per sample. Eggs were candled daily for four days. Embryos that died within 4 days were chilled at 5–6 °C for a minimum of 4–5 h before sample collection.

At 4 days post-inoculation, allantoic fluids (AF) were harvested and subjected to two-fold dilutions starting from undiluted AF. Hemagglutination assays (HA) were performed using 1% chicken red blood cells, according to the WOAH manual of diagnostic tests [21]. The titer was determined as the highest dilution showing complete hemagglutination (no streaming).

In addition, AF samples were tested for Influenza A antigen detection using a monoclonal antibody-based sandwich ELISA (MAb-NPAEL) targeting the influenza A nucleoprotein (ATCC HB65, H16-L10-4R5), as previously described [22]. The substrate solution (orthophenylenediamine 0.5 mg/mL and 0.02% H_2_O_2_ in 50 mM phosphate citrate buffer pH 5) was applied, and absorbance was measured at 492 nm using an ELISA reader (cut-off < 0.2). Two blind passages were performed before confirming the absence of viral growth.

For virus titration, 10-fold serial dilutions of milk or cheese homogenates were prepared in antibiotic-supplemented PBS. Four eggs were inoculated with 100 µL of each dilution. At 4 days post-inoculation, allantoic fluid was collected from dead embryos (≥48 h) and from live embryos. The 50% egg infectious dose (EID_50_/mL) was calculated using the Reed and Muench method.

### 2.5. Inactivation of Influenza a Virus in Ultra-High Temperature (UHT) Milk

To assess virus inactivation during a thermal treatment simulating cheese production, UHT semi-skimmed milk was spiked with the H1N1 virus (A/duck/Italy/281904-2/06). Two 300 mL batches of milk were prepared in sterile glass containers: one was spiked with 3 mL of the virus and maintained at 4–5 °C under agitation for 10 min, while the other served as a negative control. An additional sample consisting of 300 mL of PBS was spiked with 3 mL of the virus under the same conditions. Virus titration was performed on the contaminated samples (UHT milk and PBS) by calculating the EID_50_/mL.

Both milk batches were subjected to a heat treatment simulating the thermal profile of Parmigiano-Reggiano cheesemaking. The treatment consisted of holding the milk at 32 °C for 10 min, followed by 53 °C for 50 min. These steps were performed using two calibrated water baths maintained at the respective temperatures. Milk samples were placed in two identical glass beakers during heating, and temperature monitoring was conducted using a thermometer inserted in the control beaker to ensure accuracy.

Post-treatment, 10 mL samples were collected from the inoculated batch. Viral titers were quantified by SPF-ECEs to determine the 50% egg infectious dose (EID_50_/mL), according to standard protocols. Viral titers before and after heat treatment were compared to assess inactivation efficacy.

### 2.6. Cheese Production Process

#### 2.6.1. Milk and Cheese Analysis

The analyses, conducted on raw milk and cheese during the processing process, were performed at the laboratories of the IZSLER Food Safety Department, specifically with the involvement of the National Reference Center for the quality of bovine milk. The following main analytical methods were applied:

- Total bacterial count: Internal test method for the direct numbering of microorganisms in milk (total bacterial count) by opto-fluorometry—Method Accredited according to ISO 17025 [23];
- Somatic cells: Standardized test method (ISO 13366-2:2006-IDF 148-2:2006) [24] for the numbering of somatic cells in milk by opto-fluorometry—Accredited method according to ISO 17025 [23];
- Milk composition: Standardized test method (ISO 9622:2016-IDF 141) [25] for the multicomponent determination of milk production parameters by mid-infrared spectrometry—Accredited method according to ISO 17025 [23];
- Determination of freezing point: standardized test method (EN ISO 5764:2009) [26] for the determination of the freezing point of milk;
- Determination of SH acidity: Internal method test method for the de-termination of soxhlet-henkel titratable acidity in milk, cream, and some cheese-making intermediate liquids;
- Internal test method for the determination of ph in foods and solutions—Accredited Method according to ISO 17025 [23];
- Determination of the MFFB value (Moisture on Fat-Free Basis) in cheeses: to calculate the MFFB (moisture content of the defatted matter), the following formula is used.

MFFB = 100 × (100 − Fat%)/100 − Water%

The moisture content (percentage of water) is calculated by subtracting the sum of the percentages of fat, protein, fiber, carbohydrates and salt present in the cheese from 100.

#### 2.6.2. Cheesemaking Process

The cheesemaking process was conducted to replicate the traditional “Disciplinare Parmigiano-Reggiano PDO” method [27], adapted for laboratory conditions as follows:

1. Milk Collection and Skimming: raw whole cow’s milk was collected and stored chilled at no less than +8 °C. Natural skimming was performed by allowing the milk to stand for 16–18 h at 11–12 °C, enabling fat separation.
2. Starter Whey Addition: skimmed milk was supplemented with 3% (*w*/*w*) starter whey to promote acidification during subsequent steps.
3. Coagulation: the milk mixture was heated to 32 °C and held for 8–10 min to simulate the addition of rennet and the onset of curd formation.
4. Curd Cooking: the temperature was increased to 53.5 °C and maintained for 50 min to mimic curd cooking and maturation under whey.
5. Molding and Salting: the curd was transferred into molds and salted according to standard procedure.
6. Ripening: cheeses were ripened for 30 days at 6–8 °C under controlled humidity conditions.

Samples were stored in sealed containers; any manipulations that required opening the containers were carried out strictly under the biosecurity conditions described in Section 2.1. Curds were ripened in open molds, and internal cheese portions were used for virus testing.

### 2.7. Inactivation of Influenza a Virus During Grana-Type Cheese Production

#### 2.7.1. First Trial: H1N1 Virus

**Figure 1:**
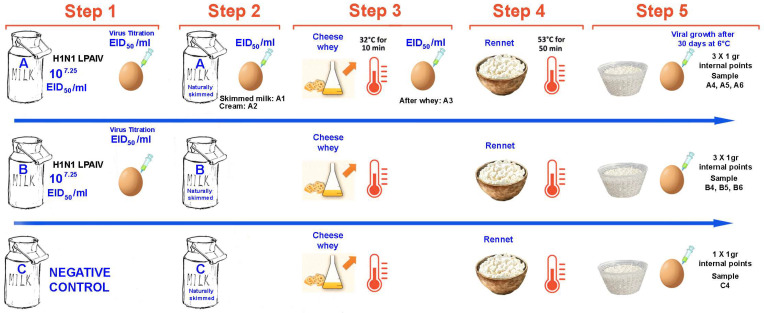
The graphs illustrate the trial 1 workflow. The phases are coded as follows: Step (1) Inoculation of 2 L raw milk with H1N1 virus; preparation of batches A and B (1 L each), and batch C (control); Step (2) Skimming; testing of skimmed milk from Batch A (A1) and cream (A2); Step (3) Addition of starter whey; sample A3 collected post-heating; Step (4) Coagulation, curd cooking (53.5 °C, 50 min), and whey maturation; Step (5) Molding, salting, and ripening; collection of three internal 1 g samples per contaminated cheese (A4–A6, B4–B6) and one internal 1 g sample per negative cheese (C4).

To assess the inactivation of influenza A virus during the production of Grana-type cheese, an initial trial was conducted using an avian H1N1 virus strain (A/duck/Italy/281904-2/06) (Figure 1). The protocol was applied to three 1 L batches of raw cow’s milk (batches A, B, and C), processed simultaneously under laboratory conditions simulating Parmigiano-Reggiano PDO cheesemaking steps.

Batches A and B were spiked with an H1N1 virus at a known titer, while batch C served as an uncontaminated negative control.

All three batches underwent the full cheesemaking procedure:

1. Step 1: Milk Collection
2. Step 2: Skimming: Natural skimming by surfacing (16–18 h at 11–12 °C);
3. Step 3: Starter Whey addition: Addition of 3% starter whey;
4. Step 4: Coagulation and curd cooking: Heating to 32 °C for 8–10 min and addition of rennet; Cooking phase at 53.5 °C for 50 min;
5. Step 5: Molding and salting: sprinkling the surface of the cheese with dry salting for 48 h
6. Step 6: Ripening at 6–8 °C for 30 days.

For batch A, samples were collected at key stages of the production process (Figure 1):

- Starter milk,
- Steps 1 and 2: Milk Collection and Skimming: Skimmed milk and cream fraction (post natural surfacing),
- Step 3: After addition of starter whey (post 32 °C pre-rennet phase)

In all collected samples, viral titers were determined by endpoint dilution assay in SPF-ECEs, and expressed as EID_50_/mL.

After ripening, three internal 1 g portions were collected from each cheese produced in batches A and B (for a total of six samples). From the negative cheese in batch C, only one internal 1 g portion was taken to assess the absence of non-specific reactions. Each sample was placed in PBS (10% *w*/*v*), and the homogenates were tested undiluted by inoculation into SPF-ECEs for viral growth detection. All eggs underwent two blind passages and were evaluated by hemagglutination assay (HA) and monoclonal antibody immunoassay (MAb-NPAEL).

#### 2.7.2. Second Trial: H5N1 Virus

**Figure 2:**
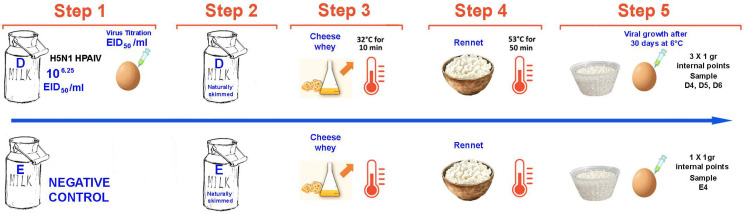
The graphs illustrate the trial 2 workflow. The phases are coded as follows: Step (1) Inoculation of 1 L raw milk with H5N1 virus; preparation of batch D and E (control); Step (2) Skimming; Step (3) Addition of starter whey; Step (4) Coagulation, curd cooking (53.5 °C, 50 min), and whey maturation; Step (5) Molding, salting, and ripening; collection of three internal 1 g samples per contaminated cheese (D4–D6) and one internal 1 g sample per negative cheese (E4).

A second trial was conducted using a high-pathogenic avian H5N1 virus (A/duck/Italy/326224-2/22, clade 2.3.4.4b) to confirm inactivation under identical cheesemaking conditions (Figure 2). Two 1 L milk batches (D and E) were processed under the biosecurity conditions described in paragraph 2.1. Viral titration was performed on initial milk, and finished cheeses were tested after 30 days of ripening. Three internal 1 g samples per contaminated cheese and one internal 1 g portion were collected and tested using SPF-ECEs and confirmatory HA and MAb-NPAEL assays. The experimental setup was analogous to the H1N1 trial.

## 3. Results

### 3.1. Preliminary Trials

The preliminary validation of the dairy matrix demonstrated the absence of toxic effects on SPF-ECEs and confirmed its ability to support viral growth. The sample spiked with H1N1 showed a higher HA titer after filtration through a 0.45 μm filter (mean: 1:512 per 25 μL) compared to the unfiltered sample (mean: 1:192 per 25 μL), based on two replicates. Filtration removes bacterial and fungal contaminants that may persist despite antibiotics, as well as coarse debris and lipid aggregates found in matrices such as cheese, which could obstruct the chorioallantoic membrane or cause mechanical irritation. Influenza A virions (approximately 80–120 nm) easily pass through the filter, so filtration does not reduce viral load but enhances the efficiency, specificity, and reproducibility of virus isolation by preventing contamination and preserving embryo viability.

Consequently, filtered samples were used for subsequent viral growth assays, while unfiltered 1:10 dilutions were used for titration in SPF-ECEs.

Virus titrations performed in parallel on PBS and UHT milk samples did not show differences in the virus titer, which averaged 10^6.75^ EID_50_/mL. Inactivation tests of the H1N1 virus in UHT semi-skimmed milk revealed a reduction in viral titer exceeding 2 log units. However, complete inactivation was not achieved, requiring the subsequent cheese production trials (Trials 1 and 2) to evaluate virus survival following the full cheesemaking and ripening process. Results are summarized in Table 1.

### 3.2. First Trial with Low-Pathogenic H1N1 Virus

In Trial 1, two liters of raw milk were placed in a sterile glass container and spiked with 25 mL of the H1N1 virus (titer: 10^7.25^ EID_50_/mL), kept at 4–5 °C under agitation for 15 min, and then divided into two batches (A and B). An additional sample of 1 L of PBS was prepared in the same way by spiking 12.5 mL of the same virus for virus titration. No differences in virus titer were detected in raw milk and PBS samples.

Only batch A underwent viral titration at different processing steps. Titrations in skimmed milk (Sample A1), cream (Sample A2), and milk post-starter whey addition (Sample A3) revealed reductions of less than 2 log_10_ units. Due to ethical considerations regarding animal use, titrations were performed from dilutions −1 to −6, sufficient for an approximate quantification of viral decrease.

After 30 days of ripening at 6–8 °C, samples from cheeses produced in batches A and B were tested. No viral growth was detected in any of the six internal 1 g cheese samples after two blind passages in SPF-ECEs, as confirmed by negative results in HA assay and MAb-NPAEL. Non-specific reactions were not detected in the sample (E4) taken from the negative cheese. Table 2 summarizes these findings.

### 3.3. Second Trial with High-Pathogenic H5N1 Virus

Trial 2 confirmed the findings of Trial 1. One 1 L batch (D) of raw milk was spiked with 10 mL of HPAIV H5N1 (clade 2.3.4.4b) in a BSL3 plus laboratory by applying the same procedure described in trial 1, under the biosecurity procedures reported in Section 2.1. Virus titration was 10^6.75^ EID_50_/mL. Batch E served as an uncontaminated negative control.

After 30 days of ripening, all internal cheese samples tested negative for virus replication, a finding further confirmed by two blind passages with negative HA and MAb-NPAEL results. Due to biosafety constraints and the objective of evaluating final virus inactivation, no intermediate titrations were performed during processing (Table 2). Given the absence of viral replication at 30 days, no additional ripening or sampling was performed.

### 3.4. Chemical and Physical Properties of the Cheeses

The raw milk used in this study originated from milk naturally destined for the production of Grana Padano PDO and had a total bacterial count of 12,000 CFU/mL and 311,000 somatic cells/mL, consistent with typical values for dairy production in Italy’s Po Valley. Its chemical composition met national standards, with fat content of 4.2 g/100 g, protein of 3.6 g/100 g, and casein of 2.85 g/100 g. Following natural skimming, the fat-to-casein ratio was adjusted to between 0.80 and 1.05, in accordance with the Parmigiano Reggiano and Grana Padano PDO specifications. Moreover, the analyses performed were those routinely conducted in the laboratory and included pH, Soxhlet–Henkel (°SH) acidity, and cryoscopy. The measured values (6.78, 3.47 °SH/50 mL, and −0.028 °C, respectively) fell within the typical ranges for milk produced in the Po Valley.

The starter whey (3% *w*/*w*), with an acidity of 31 °SH/50 mL, was sourced from a Grana Padano dairy and was used to promote proper acidification, an essential step for curd formation and subsequent maturation under whey.

The chemical–physical characterization was assessed primarily using the Moisture on Fat-Free Basis (MFFB) parameter, in accordance with the criteria established by Commission Decision 97/80/EC [28]. This parameter is considered representative of the assessment of the compliance of the product with the properties of Grana-type cheeses.

According to European classification standards, cheeses are categorized based on MFFB values as follows:

- Soft cheese: MFFB ≥ 68%;
- Semi-soft cheese: 62% ≤ MFFB < 68%;
- Semi-hard cheese: 55% ≤ MFFB < 62%;
- Hard cheese: 47% ≤ MFFB < 55%;
- Extra-hard cheese: MFFB < 47%.

The experimental protocol yielded cheeses with MFFB values between 47% and 55% after approximately 4 weeks of ripening. This characteristic classifies the products within the “hard cheese” category and is consistent with typical Grana-type cheeses such as Parmigiano-Reggiano and Grana Padano, which normally undergo maturation periods of nine months or longer. In addition, the pH and water activity (Aw) of the cheese produced in our study were evaluated after four weeks of maturation. The pH was 5.34, consistent with values reported for Parmigiano-Reggiano at the same maturation stage (between 5.28 and 5.36). The Aw value, however, was less directly comparable, as it is affected by cheese size in hard cheeses undergoing long ripening. Larger cheeses, with a lower surface-to-volume ratio, experience slower moisture loss, resulting in a more gradual reduction in Aw, particularly in the center, compared to smaller cheeses. The cheese produced in our experiment was substantially smaller in volume than a standard Parmigiano-Reggiano wheel, as it was produced under laboratory conditions. After four weeks of maturation, the Aw value in our product was <0.79, which is slightly lower than that typically observed for Parmigiano Reggiano at the end of aging (>9 months), where values range from 0.83 to 0.85. The Aw value measured at the end of our experiment is therefore consistent with that of fully matured Parmigiano Reggiano and remains below 0.9, a threshold associated with conditions unfavorable to microbial growth (Aw < 0.9). This provides additional confidence in the results obtained [29].

The successful viral inactivation observed in this study can be attributed to the combined effect of critical processing steps, including natural skimming, acidification via starter whey, curd cooking and maturation under whey, salting, and extended ripening. Together, these steps form a robust multi-barrier system that ensures the microbiological safety of the cheese, even in the absence of thermal pasteurization.

## 4. Discussion

### 4.1. Foodborne Transmission Risk of HPAIV

The global dissemination of H5N1 HPAIV, particularly widespread infection of lactating cattle in the US, has raised urgent concerns about the potential risk of zoonotic transmission through dairy products.

Although human infections were historically associated mainly to direct contact with infected birds, mammals, or contaminated environments, the detection of high viral loads in the milk from infected cattle highlights the need to assess foodborne transmission risks, particularly through the consumption of unpasteurized milk and dairy products.

Key risk factors include: (i) the infectious viral load in milk; (ii) virus stability under refrigeration; (iii) efficacy of processing steps in reducing infectivity; and (iv) host susceptibility, particularly the minimal infectious dose [30]. H5N1 has been shown to preferentially target bovine mammary tissue, where both avian-type (α2,3-linked) and human-type (α2,6-linked) sialic acid receptors are expressed, facilitating high-level viral shedding into milk [9,31]. Furthermore, the virus can remain viable in refrigerated milk (4 °C) for over five weeks [32], underscoring the importance of downstream processing in mitigating transmission risk.

### 4.2. Impact of Cheesemaking Processes on Virus Inactivation

The implementation of effective milk processing and production protocols is essential to ensure the inactivation of viruses prior to the consumption of dairy products. Pasteurization remains the gold standard for inactivating avian influenza viruses (AIVs) in milk and dairy products, as consistently demonstrated in previous studies [33,34,35]. While viral RNA has occasionally been detected in thermally treated products such as butter, cheese, and ice cream, no viable virus has been recovered, confirming the efficacy of standard pasteurization protocols in inactivating AIVs. However, the fate of highly pathogenic avian influenza viruses (HPAIVs) during the manufacture of raw-milk cheeses has been largely unexplored [36].

Our study addressed this critical knowledge gap by experimentally assessing the inactivation of both low-pathogenic (H1N1) and highly pathogenic (H5N1 clade 2.3.4.4b) influenza A viruses during the production of Grana-type hard cheese from raw cow’s milk under conditions conforming to Parmigiano-Reggiano PDO specifications. Cheese samples were tested in triplicate after 30 days of ripening to assess residual viral infectivity. Across both experimental arms, no viable virus was detected in any sample, indicating complete inactivation under the applied production conditions. Given that these results were consistent and conclusive, the ripening period was not extended further.

This outcome is especially relevant given that the initial viral titers employed in our experiments (10^7.25^ EID_50_/mL for H1N1 and 10^6.75^ EID_50_/mL for H5N1) exceeded both the mean and maximum viral loads reported in naturally contaminated milk samples from recent U.S. outbreaks (mean: 10^3.5^ EID_50_/mL; max: 10^6.3^ EID_50_/mL) [32]. Furthermore, regulatory frameworks require the exclusion of visibly altered milk from processing, thereby further minimizing the potential risk of consumer exposure. These considerations strengthen the relevance and applicability of our findings.

This study prioritized evaluating focused on assessing the absence of viral replication in the final cheese rather than measuring viral titers during intermediate processing stages, which are of limited relevance to consumer food safety. Moreover, previous contamination studies [37] evaluating the inactivation of pathogenic bacteria in raw milk intended for the production of “Grana” cheese demonstrated that the skimming stage functions as a barrier to the transfer of biological agents from milk to cheese only in the presence of spore-forming microorganisms (e.g., *Clostridium* spp., *Bacillus* spp.) or bacteria of the genus Mycobacterium (e.g., *Mycobacterium avium* subsp. *paratuberculosis*). This effect is likely attributable to their relatively large size, which facilitates their removal from milk through natural skimming. In the present study, considering the small size of the viral particle, it was reasonable to assume that any barrier effect during intermediate stages would be negligible. Consequently, the titrations performed after these stages, conducted exclusively on contaminated batch A, served primarily to corroborate our hypothesis of a limited reduction in viral titer. However, these results are only indicative, as sampling was limited to a single batch with one sample per step; additional sampling is required to provide more robust conclusions.

In addition, the Production Regulations of Parmigiano-Reggiano PDO establish a stringent framework of rules and standards that must be followed by all authorized dairies throughout the entire cheesemaking process. Only producers affiliated with the Parmigiano-Reggiano Consortium, founded in 1934, are authorized to manufacture this iconic Italian cheese under the PDO label. The Consortium is responsible for safeguarding, supervising, enhancing, promoting, and internationally representing Parmigiano-Reggiano, ensuring both the quality of the product and the integrity of its production process. By establishing the technological procedures and compositional standards for the final product, the Consortium guarantees uniformity across all certified producers. This standardization minimizes variability and ensures that cheeses labeled as Parmigiano-Reggiano consistently meet the high safety and quality benchmarks established by the PDO specification.

### 4.3. Comparison with Literature

In our study, artificial milk contamination was used. Spiking experiments in food products, particularly milk and dairy products, are explicitly recognized in relevant guidelines as an appropriate method for testing the inactivation of microorganisms [38]. This approach is often necessary in many cases because naturally contaminated milk is not available, ensuring that studies can be performed with the required levels of repeatability and reproducibility. This is particularly true in the European Union, where no naturally contaminated milk has been detected; therefore, European-type cheese cannot be investigated without the use of artificially contaminated milk.

Indeed, numerous published studies have adopted laboratory-based milk contamination methods [17,34,36,39], which allow precise control over viral or bacterial titers. The guidelines published by Condron et al. [38] recommend using artificial contamination with titers representative of natural conditions, which is exactly the approach we followed. Several recent studies have employed the artificial contamination of milk under laboratory conditions, consistent with the methodological approach used in our investigation.

Furthermore, milk containing a high H5N1 viral load most likely comes from animals with clinical mastitis, a condition that alters the appearance and composition of the milk. Milk of this type is unsuitable for cheese production under standard conditions and is usually excluded from processing. Therefore, in many cases, it is difficult to use milk with high viral loads resulting from natural infections, especially in bulk milk.

In the H1N1 trial, intermediate titration after skimming, cream separation, and whey addition revealed ~1 log reduction in viral load, confirming that these steps alone do not fully inactivate the virus. This aligns with prior reports indicating that moderate heat treatment (e.g., 50–54 °C) is insufficient to ensure complete viral inactivation [32,33,34].

While pasteurization remains the gold standard for eliminating viral pathogens in milk, our findings support that the integrated raw-milk cheesemaking process, including curd cooking above 52 °C, whey fermentation, and extended ripening, can similarly achieve complete inactivation of AIV.

Our results are consistent with those of Nooruzzaman et al. [36], who reported that viable H5N1 could be recovered from raw-milk cheeses with pH ≥ 5.8, but not from those acidified to pH 5.0 after 60 days of ripening. To date, that study is the only other investigation of H5N1 survival in raw-milk cheese. The final pH in commercial Parmigiano-Reggiano cheese (~5.3) as well as in our study (5.34) was closer to this inactivation threshold, underscoring the critical role of acidification in virus control. Furthermore, another study [34] showed that thermization (heat treatment below pasteurization) of raw milk at temperatures above 54 °C successfully inactivates the virus within 15 min, offering an alternative safety measure for cheese production. In our study, the milk used in the production of Parmigiano Reggiano cheese was subjected to a heat profile of 53 °C for 50 min, which is very close to 54 °C but significantly longer in duration. These data further reinforce the results obtained in our study.

The calculated MFFB and pH values confirmed that the final product was representative of authentic Parmigiano-Reggiano PDO cheese. Our Aw value was obviously lower than that of Parmigiano-Reggiano because it depends on the volume of the cheese. However, in PDO-compliant production, Aw decreases progressively during maturation, reaching values similar to ours at the end of the minimum maturation period, creating adverse conditions for viral persistence. It is widely known that Aw values below 0.85 create an environment unfavorable for the growth of microorganisms, and this parameter is commonly used to assess microbiological safety in food products. Some published studies [40,41] have examined the effect of this parameter on enveloped viruses, such as avian influenza viruses, by investigating their persistence under various environmental conditions. The decay rate constants of enveloped viruses are influenced by factors such as relative humidity and water activity, indicating that lower water activity accelerates viral inactivation. Enveloped viruses possess a lipid bilayer membrane that is crucial for their infectivity, and their stability is significantly affected by environmental conditions, including water activity. Although our experiments did not encompass the full 9-month ripening period specified for Grana PDO cheeses, complete viral inactivation was observed by day 30. Considering that additional reductions in pH and Aw occur throughout ripening, it is highly improbable that viable virus would persist in cheese aged in compliance with PDO requirements.

Moreover, the use of two distinct influenza A viruses, one highly pathogenic and one low pathogenic, yielding identical results supports the hypothesis that influenza A viruses share a similar fate during cheese maturation. These findings provide strong evidence for the safety of cheese, even in scenarios where other influenza viruses may circulate and pose potential future concerns.

## 5. Conclusions

This study provides robust experimental evidence that traditional Grana-type cheesemaking with raw milk, as defined by the Parmigiano-Reggiano PDO specification, ensure complete inactivation of both low-pathogenic (H1N1) and highly pathogenic (H5N1 clade 2.3.4.4b) avian influenza viruses. Even when cheeses were produced using inocula that far exceeded the viral loads reported during natural epidemics, no infectious virus was detected after 30 days of aging, demonstrating a substantial safety margin beyond real-world contamination scenarios.

The combined effects of curd cooking, acidification, whey removal, salting, and prolonging ripening provide multiple sequential barriers to inactivation that reliably eliminate AIV infectivity. Conducting the experiments in strict compliance with PDO production specifications reinforces the reproducibility and real-world relevance of the results and their transferability to commercial cheesemaking.

Overall, the results provide strong assurance that Grana-type cheese production processes, when performed correctly, render hard cheeses made from raw milk safe, even in the unlikely event of HPAIV contamination at the farm level. These data fill a critical gap in the evidence available to risk assessors and support the conclusion that the risk of foodborne transmission of HPAIV through such cheeses is negligible. Furthermore, the study highlights the essential role of regulated processing parameters, in particular temperature, pH development, and ripening duration, as effective and reliable control measures that contribute to the protection of public health.

## Figures and Tables

**Table 1:** Summary of viral titration in UHT milk following heat treatment.

Trial	Virus	Virus Titer in Milk (10^x^ EID_50_/mL)	Batch	Heat Treatment	Milk After Heat Treatment (10^x^ EID_50_/mL)
UHT milk	A/duck/Italy/281904-2/06 H1N1	6.75	UHT 1	32 °C for 10 min; 53 °C for 50 min	4.00
UHT milk	NO	Nt	UHT 2	-	Nt

Nt, Not tested.

**Table 2:** Summary of virus titration and growth assays during cheese production trials.

Trial	Virus	Production Phases					
		Batch	Milk	Skimmed Milk	Cream	After Starter Whey	Cheese After 30 Days Ripening
			Virus Titer (10^x^ EID_50_/mL)	Virus Titer (10^x^ EID_50_/mL)	Virus Titer (10^x^ EID_50_/mL)	Virus Titer (10^x^ EID_50_/mL)	Virus Growth Determination (Internal Portions of 1 g)
		Samples		1	2	3	4, 5, 6
1	A/duck/Italy/281904-2/06 (H1N1)	A	7.25	Sample A1 > 5.00	Sample A2 > 5.00	Sample A3 = 6.00	A4: HA < 1; Mab-NPAEL AV: 0.10—Negative
							A5: HA < 1; Mab-NPAEL AV: 0.08—Negative
							A6: HA < 1; Mab-NPAEL AV: 0.09—Negative
		B	7.25	Nt	Nt	Nt	B4: HA < 1; Mab-NPAEL AV:0.11—Negative
							B5: HA < 1; Mab-NPAEL AV: 0.13—Negative
							B6: HA < 1; Mab-NPAEL AV: 0.08—Negative
	NO	C	NO	Nt	Nt	Nt	C4: HA < 1; Mab-NPAEL AV: 0.08—Negative
2	A/duck/Italy/326224-2/22 (H5N1)	D	6.75	Nt	Nt	Nt	D4: HA < 1; Mab-NPAEL AV: 0.12—Negative
							D5: HA < 1; Mab-NPAEL AV: 0.09—Negative
							D6: HA < 1; Mab-NPAEL AV: 0.07—Negative
	NO	E	NO	Nt	Nt	Nt	E4: HA < 1; Mab-NPAEL AV: 0.08—Negative

The samples were labeled with the batch letter followed by a number according to the step as shown in Figure 1 and Figure 2. Negative = no viral growth detected after two blind passages; Nt: Not tested. HA titer/25 μL; Absorbance value (AV) (492 nm): cut-off value 0.2.

## Data Availability

Data available upon request.

## References

[1] Charostad J., Rukerd M.R.Z., Mahmoudvand S., Bashash D., Hashemi S.M.A., Nakhaie M., Zandi K. (2023). A comprehensive review of highly pathogenic avian influenza (HPAI) H5N1: An imminent threat at doorstep. Travel Med. Infect. Dis..

[2] Caliendo V., Lewis N.S., Pohlmann A., Baillie S.R., Banyard A.C., Beer M., Brown I.H., Fouchier R.A.M., Hansen R.D.E., Lameris T.K. (2022). Transatlantic spread of highly pathogenic avian influenza H5N1 by wild birds from Europe to North America in 2021. Sci. Rep..

[3] Burrough E.R., Magstadt D.R., Petersen B., Timmermans S.J., Gauger P.C., Zhang J., Siepker C., Mainenti M., Li G., Thompson A.C. (2024). Highly pathogenic avian influenza A(H5N1) clade 2.3.4.4b virus infection in domestic dairy cattle and cats, United States, 2024. Emerg. Infect. Dis..

[4] Sreenivasan C.C., Thomas M., Kaushik R.S., Wang D., Li F. (2019). Influenza A in Bovine Species: A Narrative Literature Review. Viruses.

[5] Centers for Disease Control and Prevention Bird Flu in Dairy Cows: Current Situation. CDC. https://www.aphis.usda.gov/livestock-poultry-disease/avian/avian-influenza/hpai-detections/hpai-confirmed-cases-livestock.

[6] Caserta L.C., Frye E.A., Butt S.L., Laverack M., Nooruzzaman M., Covaleda L.M., Thompson A.C., Koscielny M.P., Cronk B., Johnson A. (2024). Spillover of highly pathogenic avian influenza H5N1 virus to dairy cattle. Nature.

[7] Mostafa A., Nogales A., Martinez-Sobrido L. (2025). Highly pathogenic avian influenza H5N1 in the United States: Recent incursions and spillover to cattle. Npj Viruses.

[8] Oguzie J.U., Marushchak L.V., Shittu I., Lednicky J.A., Miller A.L., Hao H., Nelson M.I., Gray G.C. (2024). Avian Influenza A(H5N1) Virus among Dairy Cattle, Texas, USA. Emerg. Infect. Dis..

[9] Halwe N.J., Cool K., Breithaupt A., Schön J., Trujillo J.D., Nooruzzaman M., Kwon T., Ahrens A.K., Britzke T., McDowell C.D. (2025). H5N1 clade 2.3.4.4b dynamics in experimentally infected calves and cows. Nature.

[10] Centers for Disease Control and Prevention (CDC) H5 Bird Flu: Current Situation. https://www.cdc.gov/bird-flu/situation-summary/index.html#human-cases.

[11] Food and Agriculture Organization of the United Nations (FAO) Global Avian Influenza Viruses with Zoonotic Potential Situation Update. https://www.fao.org/animal-health/situation-updates/global-aiv-with-zoonotic-potential/en.

[12] World Health Organization (WHO) Cumulative Number of Confirmed Human Cases for Avian Influenza A(H5N1) Reported to WHO, 2003–2025, 20 January 2025. https://www.who.int/publications/m/item/cumulative-number-of-confirmed-human-cases-for-avian-influenza-a(h5n1)-reported-to-who--2003-2025--20-january-2025.

[13] Alexakis L., Buczkowski H., Ducatez M., Fusaro A., Gonzales J.L., Kuiken T., Ståhl K., European Food Safety Authority (EFSA), European Centre for Disease Prevention and Control (ECDC), European Union Reference Laboratory for Avian Influenza (EURL) (2025). Scientific report: Avian influenza overview December 2024–March 2025. EFSA J..

[14] Food and Drug Administration (FDA) Milk Safety Program & Shippers List. https://www.fda.gov/food/federal-state-local-tribal-and-territorial-cooperative-human-food-programs/milk-safety-program-shippers-list.

[15] U. S. Department of Health and Human Services (HHS) Grade “A” Pasteurized Milk Ordinance, Including 1372 Provisions from the Grade “A” Condensed and Dry Milk Products and Condensed and Dry Whey—1373 Supplement I to the Grade “A” Pasteurized Milk Ordinance. https://www.fda.gov/media/140394/download.

[16] Spackman E., Anderson N., Walker S., Suarez D.L., Jones D.R., McCoig A., Colonius T., Roddy T., Chaplinski N.J. (2024). Inactivation of Highly Pathogenic Avian Influenza Virus with High-temperature Short Time Continuous Flow Pasteurization and Virus Detection in Bulk Milk Tanks. J. Food Prot..

[17] Lang J., Helke D., Kuryshko M., Abdelwhab E.M. (2024). Survivability of H5N1 Avian Influenza Virus in Homemade Yogurt, Cheese and Whey. Emerg. Microbes Infect..

[18] Spackman E., Senne D.A., Myers T.J., Bulaga L.L., Garber L.P., Perdue M.L., Lohman K., Daum L.T., Suarez D.L. (2002). Development of a real-time reverse transcriptase PCR assay for type A influenza virus and the avian H5 and H7 hemagglutinin subtypes. J. Clin. Microbiol..

[19] U.S. Food and Drug Administration (FDA) (2023). Extraction and Detection of Influenza A Virus in Milk and Milk Products.

[20] (2017). Microbiology of the Food Chain—Horizontal Method for Determination of Hepatitis A Virus and Norovirus Using Real-Time RT-PCR—Part 1: Method for Quantification.

[21] World Organization for Animal Health (WOAH) (2024). Avian Influenza (Infection with Avian Influenza Viruses). Manual of Diagnostic Tests and Vaccines for Terrestrial Animals.

[22] Moreno A., Chiapponi C., Boniotti M.B., Sozzi E., Foni E., Barbieri I., Zanoni M.G., Faccini S., Lelli D., Cordioli P. (2012). Genomic characterization of H1N2 swine influenza viruses in Italy. Vet. Microbiol..

[23] (2017). General Requirements for the Competence of Testing and Calibration Laboratories.

[24] (2006). Milk—Enumeration of Somatic Cells—Part 2: Guidance on the Operation of Fluoro-Opto-Electronic Counters.

[25] (2016). Milk and Liquid Milk Products—Guidelines for the Application of Mid-Infrared Spectrometry.

[26] (2009). Milk—Determination of Freezing Point—Thermistor Cryoscope Method (Reference Method).

[27] Consorzio del Formaggio Parmigiano Reggiano Disciplinare di Produzione Della Denominazione di Origine Protetta “Parmigiano Reggiano”. https://www.parmigianoreggiano.com.

[28] The Commission of the European Communities (1997). 97/80/EC: Commission Decision of 18 December 1996 Laying down Provisions for the Implementation of Council Directive 96/16/EC on Statistical Surveys of Milk and Milk Products (Text with EEA Relevance).

[29] Gomez E.J., Delgado J.A., Gonzalez J.M. (2021). Influence of water availability and temperature on estimates of microbial extracellular enzyme activity. PeerJ.

[30] Owusu H., Sanad Y.M. (2025). Comprehensive Insights into Highly Pathogenic Avian Influenza H5N1 in Dairy Cattle: Transmission Dynamics, Milk-Borne Risks, Public Health Implications, Biosecurity Recommendations, and One Health Strategies for Outbreak Control. Pathogens.

[31] Nelli R.K., Harm T.A., Siepker C., Groeltz-Thrush J.M., Jones B., Twu N.C., Nenninger A.S., Magstadt D.R., Burrough E.R., Piñeyro P.E. (2024). Sialic Acid Receptor Specificity in Mammary Gland of Dairy Cattle Infected with Highly Pathogenic Avian Influenza A(H5N1) Virus. Emerg. Infect. Dis..

[32] Spackman E., Jones D.R., Mc Coig A.M., Colonius T.J., Goraichuk I.V., Suarez D.L. (2024). Characterization of highly pathogenic avian influenza virus in retail dairy products in the US. J. Virol..

[33] Martin N.H., Trmcic A., Alcaine S.D. (2024). Hot topic: Avian influenza subtype H5N1 in US dairy—A preliminary dairy foods perspective. JDS Commun..

[34] Nooruzzaman M., Covaleda L.M., de Oliveira P.S.B., Martin N.H., Koebel K.J., Ivanek R., Alcaine S.D., Diel D.G. (2025). Thermal inactivation spectrum of influenza A H5N1 virus in raw milk. Nat. Commun..

[35] Suarez D.L., Goraichuk I.V., Killmaster L., Spackman E., Clausen N.J., Colonius T.J., Leonard C.L., Metz M.L. (2025). Testing of Retail Cheese, Butter, Ice Cream, and Other Dairy Products for Highly Pathogenic Avian Influenza in the US. J. Food Prot..

[36] Nooruzzaman M., de Oliveira P.S.B., Butt S.L., Martin N.H., Alcaine S.A., Walker S.P., Diel D.G. (2025). H5N1 influenza virus stability and transmission risk in raw milk and cheese. Nat. Med..

[37] D’Incecco P., Faoro F., Silvetti T., Schrader K., Pellegrino L. (2015). Mechanisms of Clostridium tyrobutyricum Removal through Natural Creaming of Milk: A Microscopy Study. J. Dairy Sci..

[38] Condron R., Farrokh C., Jordan K., McClure P., Ross T., Cerf O. (2015). Guidelines for Experimental Design Protocol and Validation Procedure for the Measurement of Heat Resistance of Microorganisms in Milk. Int. J. Food Microbiol..

[39] Blondin-Brosseau M., Zhang W., Gravel C., Harlow J., Li X., Nasheri N. (2025). Comparison of Methods for Extraction of Infectious Influenza Virus from Raw Milk Cheeses. J. Food Prot..

[40] Roos Y.H. (2020). Water and Pathogenic Viruses Inactivation-Food Engineering Perspectives. Food Eng. Rev..

[41] Silverman A.I., Boehm A.B. (2021). Systematic Review and Meta-Analysis of the Persistence of Enveloped Viruses in Environmental Waters and Wastewater in the Absence of Disinfectants. Environ. Sci. Technol..

